# Mental Health Indicators in Older Men Practising Aquatic Exercise and Multicomponent Exercise: An Exploratory Cross-Sectional Study

**DOI:** 10.3390/healthcare14193343

**Published:** 2026-10-08

**Authors:** Carlos Farinha, José Pedro Ferreira, Ana Teixeira, Samuel Honório, Marco Batista, Jorge Santos, Miguel Rebelo, João Petrica, João Serrano

**Affiliations:** 1 Department of Sports and Well-Being, Polytechnic University of Castelo Branco, 6000-266 Castelo Branco, Portugal; samuelhonorio@ipcb.pt (S.H.); marco.batista@ipcb.pt (M.B.); jorgesantos@ipcb.pt (J.S.); miguel.rebelo@ipcb.pt (M.R.); j.petrica@ipcb.pt (J.P.); j.serrano@ipcb.pt (J.S.); 2 SPRINT Sport Physical Activity and Health Research & Innovation Center, 6000-084 Castelo Branco, Portugal; 3 Faculty of Sport Sciences and Physical Education, University of Coimbra, 3040-248 Coimbra, Portugal; jpferreira@fcdef.uc.pt (J.P.F.); ateixeira@fcdef.uc.pt (A.T.); 4 CIPER—Interdisciplinary Center for the Study of Human Performance, Faculty of Sport Sciences and Physical Education, University of Coimbra, 3040-248 Coimbra, Portugal; 5 Piaget Research Center for Ecological Human Development, Instituto Piaget, 1950-157 Lisboa, Portugal

**Keywords:** ageing, aquatic exercise, multicomponent exercise, mental health, life satisfaction, older men

## Abstract

Background/Objectives: Regular physical exercise is considered a relevant strategy for promoting physical and mental health in older adults. However, studies using exclusively male samples that directly compare different exercise modalities in mental health indicators remain scarce. Therefore, this study aimed to compare mental health indicators between older men practising aquatic physical exercise and older men practising multicomponent physical exercise. Methods: This exploratory cross-sectional study included 17 older men, divided into two groups: aquatic exercise (AE; n = 8; 70.8 ± 4.5 years) and multicomponent exercise (ME; n = 9; 75.4 ± 4.1 years). We assessed the following mental health indicators: self-esteem (Rosenberg Self-Esteem Scale); life satisfaction (Satisfaction with Life Scale); depressive symptoms (Geriatric Depression Scale); and perceived stress (Perceived Stress Scale). As sample characterisation variables, body composition parameters were analysed: body weight, bioelectrical impedance-derived visceral fat level, percentage of fat mass, and percentage of muscle mass. Results: In the unadjusted parametric analysis, life satisfaction differed significantly between groups, with higher values in the ME group (*p* = 0.029; *d* = −1.174; 95% CI: −2.20 to −0.12). However, this difference no longer reached the threshold for statistical significance after the Bonferroni correction for multiple comparisons (adjusted *p* = 0.116) or in the non-parametric sensitivity analysis (*p* = 0.054). We observed no statistically significant between-group differences in self-esteem, depressive symptoms, or perceived stress. Conclusions: In this sample of older men, the unadjusted parametric analysis showed a between-group difference in life satisfaction, with higher values in the multicomponent exercise group. However, this finding did not remain statistically significant after correction for multiple comparisons or in the non-parametric sensitivity analysis. No statistically significant between-group differences were detected in the remaining mental health indicators analysed. Nevertheless, given the small sample size and limited statistical power, the absence of statistically significant differences in these indicators should not be interpreted as evidence of similarity between the exercise modalities. These findings should be considered exploratory and interpreted with caution, also taking into account the cross-sectional design of the study.

## 1. Introduction

Ageing is associated with physical, functional, and psychological changes that may influence mental health and quality of life in older adults. In this context, regular physical exercise is recommended as an important strategy to promote healthy ageing, contributing not only to maintaining functional capacity but also to psychological well-being [1]. Recent evidence has shown that physical exercise is associated with better mental health indicators in older adults, including fewer depressive symptoms, lower anxiety, better perceived well-being, and greater social participation [2,3].

Among the exercise modalities frequently used in this population, aquatic exercise stands out due to the specific characteristics of the water, such as buoyancy and reduced joint impact, which may favour adherence and participation among older adults with different levels of physical fitness [4]. Recent studies suggest that aquatic exercise may positively affect mood and anxiety symptoms, although further studies with robust methodological designs are still needed [5].

On the other hand, multicomponent exercise programmes, which integrate different physical capacities such as strength, endurance, balance, and flexibility, are also widely used in older adults. Research has shown that this type of programme may improve physical function, cognitive capacity, frailty, and some psychological indicators, including depressive symptoms [6]. 

Additionally, body composition variables, including fat mass, muscle mass, and visceral fat, were considered in order to provide a more comprehensive physical characterisation of the participants. Although changes in body composition have been associated with a risk of depression and a decline in overall health status [7], these variables were used in the present study solely for sample characterisation. They were not considered as covariates or explanatory variables in the main analyses.

Conceptually, aquatic exercise and multicomponent exercise have distinct characteristics that may relate differently to dimensions of physical and psychological health in older adults. Aquatic exercise is performed in a low-impact environment with specific physiological and mechanical characteristics and may improve different components of physical function, including balance, muscle strength, flexibility, and cardiorespiratory fitness [8]. In parallel, systematic reviews indicate that aquatic exercise may also be associated with improvements in mood, anxiety, and depressive symptoms, although the available evidence remains heterogeneous [4].

In turn, multicomponent exercise programmes combine different types of stimuli, typically including aerobic, strength, and balance components, to simultaneously target different dimensions of functional fitness. Recent evidence indicates that this type of exercise can meaningfully improve functional fitness in older adults and may also reduce depressive symptoms. However, considerable heterogeneity and limited certainty of evidence remain in this latter domain [9,10]. These distinct characteristics may, theoretically, provide different experiences in terms of physical competence, functional capacity, autonomy, and well-being and may, through these pathways, relate differently to indicators such as self-esteem, life satisfaction, depressive symptoms, and perceived stress. However, these mechanisms should be regarded as conceptual hypotheses rather than as established relationships between each exercise modality and specific psychological outcomes.

Recent evidence further supports exploring differences between exercise modalities, suggesting that the type of physical activity practised may be associated with differences in mental health and social integration indicators in older adults [11]. However, direct comparative evidence between aquatic exercise and multicomponent exercise regarding the psychological indicators considered in the present study remains limited. For this reason, and given the lack of consistent evidence to predict the superiority of either modality or to identify a particularly responsive outcome, the present study retained an exploratory design, and no specific directional hypothesis was formulated for any of the four prespecified mental health outcomes.

Despite the relevance of these two modalities, studies with exclusively male samples that directly compare aquatic exercise practitioners and multicomponent exercise practitioners in mental health-related variables remain scarce [4,5]. This approach is relevant, as the relationship between physical activity and depressive symptoms may differ between older men and women [8].

Therefore, the present study aimed to compare mental health indicators between older men practising aquatic exercise and older men practising multicomponent exercise.

## 2. Materials and Methods

### 2.1. Study Design

An exploratory cross-sectional study was conducted with 17 older men, divided into two groups according to the exercise modality practised: aquatic exercise (AE; n = 8; 70.8 ± 4.5 years) and multicomponent exercise (ME; n = 9; 75.4 ± 4.1 years). Four mental health outcomes were prespecified as co-primary outcomes: self-esteem, assessed using the Rosenberg Self-Esteem Scale (RSES); life satisfaction, assessed using the Satisfaction with Life Scale (SWLS); depressive symptoms, assessed using the Geriatric Depression Scale (GDS); and perceived stress, assessed using the Perceived Stress Scale (PSS). As secondary variables used exclusively for sample characterisation, body composition parameters were analysed, namely body weight (W), bioelectrical impedance-derived visceral fat level (VF), percentage of fat mass (FM), and percentage of muscle mass (MM). These variables were not considered as covariates, moderators, or explanatory variables in the main analyses. All variables were assessed at a single time point. The statistical analysis was performed using coded datasets, without direct identification of the participants. The present study was conducted in accordance with the ethical principles of the Declaration of Helsinki and was approved by the Ethics Committee of the Faculty of Sport Sciences and Physical Education, University of Coimbra (reference: CE/FCDEF-UC/00462019). All participants were informed about the study aims and procedures and provided written informed consent before data collection. Anonymity, data confidentiality, and the right to withdraw from participation at any time without any penalty were guaranteed.

### 2.2. Participants

Participants were recruited from the Central region of Portugal using a convenience sample. Participants were recruited, and data were collected during May 2018. The following inclusion criteria were considered: male sex; age of 60 years or older; non-institutionalised individuals; regular participation, for at least 6 months, in supervised aquatic exercise programmes or multicomponent exercise programmes; and voluntary acceptance to participate in the study, formalised by signing an informed consent form. Exclusion criteria were attendance below 75% of the total number of sessions over the previous 6 months and inability to complete the assessment procedures planned for the study. Seventeen potentially eligible participants were approached, and all agreed to participate in the study. No refusals or exclusions occurred after assessing the eligibility criteria.

### 2.3. Exercise Programmes

Participants were enrolled in two regular physical exercise programmes, differentiated by the environment in which they were performed and by the predominant characteristics of the modality.

The aquatic exercise (AE) group comprised participants enrolled in an aquatic gymnastics programme conducted in a heated indoor swimming pool. The sessions followed the following structure: 10 min of joint mobilisation, muscle activation, and adaptation to the aquatic environment; 30 min of predominantly aerobic exercises, including locomotor movements, simple choreographed routines, and aquatic gymnastics exercises, performed with and without auxiliary equipment and to music at a tempo of 130–140 beats per minute; and 5 min of cool-down and stretching.

The multicomponent exercise (ME) group comprised participants enrolled in a senior exercise programme conducted on land. Sessions consisted of three parts: 10 min of joint mobilisation and muscle activation; 30 min of exercises aimed at developing several components of functional fitness, including muscle strength, balance, coordination, flexibility, and aerobic endurance; and 5 min of cool-down and stretching.

In both groups, participants had been practising their respective exercise modality for at least six months in a setting supervised by qualified Sport Sciences professionals. They were integrated into mixed-sex classes comprising both men and women. Sessions lasted 45 min and were conducted twice weekly. The mean attendance rate was 85.49% (AE: 84.40%; ME: 86.58%). Exercises were adapted to the participants’ functional characteristics and individual limitations, with progression individualised according to the tolerance and comfort demonstrated during the sessions. The target intensity ranged from 4 to 6 on the Borg Category-Ratio 10 (CR10) Rating of Perceived Exertion scale [12] and was monitored by the professionals responsible for the sessions. Different professionals supervised the exercise sessions in the two programmes.

### 2.4. Instruments

#### 2.4.1. Body Composition Parameters

The same previously trained researcher measured all body composition parameters. The following parameters were assessed: body weight (W), bioelectrical impedance-derived visceral fat level (VF), percentage of fat mass (FM), and percentage of muscle mass (MM). All measurements were taken using a TANITA BC-601 electrical bioimpedance scale (Tanita Corporation, Tokyo, Japan), following the manufacturer’s instructions.

Body composition assessments were performed in the morning, at approximately the same time for all participants, and always before the corresponding exercise session. Participants were encouraged to avoid alcohol and caffeine consumption before the assessment and to empty their bladder before measurement. However, no specific instructions were provided regarding fasting or hydration status.

#### 2.4.2. Mental Health Indicators

The mental health indicators analysed were self-esteem, life satisfaction, depressive symptoms, and perceived stress, assessed using the following scales validated for the Portuguese population:

- Self-esteem was assessed using the Rosenberg Self-Esteem Scale (RSES) [13]. This instrument assesses global self-esteem and consists of 10 items, answered on a 4-point Likert scale. The total score was calculated by summing the items, after reversing the negatively worded items, according to the instructions of the version used. Scores range from 10 to 40, with higher scores indicating higher self-esteem.
- Life satisfaction was assessed using the Satisfaction with Life Scale (SWLS) [14]. This instrument assesses global life satisfaction and consists of 5 items answered on a 7-point Likert scale. The final score ranges from 5 to 35 points, with higher scores indicating greater life satisfaction.
- Depressive symptoms were assessed using the short 15-item version of the Geriatric Depression Scale (GDS) [15]. This instrument is used to screen for depressive symptoms in older adults and has a dichotomous response format, with “yes” or “no” answers. The total score was calculated according to the instructions of the version used. Scores between 0 and 5 points indicate the absence of relevant signs of depression, scores between 6 and 10 points suggest mild to moderate depressive symptoms, and scores between 11 and 15 points suggest more pronounced depressive symptoms.
- Perceived stress was assessed using the Perceived Stress Scale (PSS) [16,17]. This instrument assesses perceived stress during the previous month and consists of 10 items answered on a 5-point Likert scale. The final score ranges from 0 to 40 points, with higher scores indicating higher levels of perceived stress.

### 2.5. Data Collection Procedure

Data collection was carried out in two distinct stages. During a first visit to the facilities where the classes took place, the study was presented to the participants, and its aims, procedures, and participation conditions were explained. After this clarification, the researchers obtained written informed consent.

In a second stage, on the day of the following session, data on body composition parameters and mental health indicators were collected. Assessments were carried out individually in a room prepared for this purpose to ensure participants’ privacy. All participants were assessed under the same conditions and by the same research team. The assessors were aware of the exercise group to which each participant belonged. All questionnaires were completed in full, with no missing responses.

### 2.6. Statistical Analysis

Given the exploratory nature of the study and the limited availability of eligible participants, we did not perform an a priori sample size calculation. Subsequently, a sensitivity analysis was conducted using G*Power version 3.1.9.6 [18,19], considering two independent groups (AE: n = 8; ME: n = 9), α = 0.05, and statistical power of 80%. This analysis indicated that the study design would detect only very large effects, approximately Cohen’s *d* = 1.46. Therefore, the results should be interpreted with caution, considering effect magnitude alongside the *p*-value.

The statistical analysis included descriptive statistics, presenting the mean, standard deviation, minimum values, and maximum values. The normality of data distribution was assessed using the Shapiro–Wilk test, kurtosis analysis, and visual inspection of histograms. Homogeneity of variances was analysed using Levene’s test. The presence of potential outliers was assessed through visual inspection of boxplots. The identified values were subsequently verified in the database and retained in the analyses, as they did not result from data collection or data entry errors.

For between-group comparisons, an independent-samples Student’s *t*-test was used for variables for which the assumption of homogeneity of variances was met. For variables for which this assumption was not met, Welch’s *t*-test was used. Effect size magnitude was calculated using Cohen’s *d* and classified as trivial (<0.20), small (0.20–0.49), moderate (0.50–0.79), or large (≥0.80), according to the criteria proposed by Cohen (1988 [20]).

Because four co-primary mental health outcomes were prespecified, we first performed unadjusted between-group analyses to estimate the magnitude and direction of observed differences for each outcome. However, because multiple co-primary outcomes were tested, we applied a Bonferroni correction to control inflation of the Type I error rate. Accordingly, the primary inferential interpretation of statistical significance across the four co-primary outcomes was based on the multiplicity-adjusted results, while the unadjusted results were retained as complementary exploratory information.

Additionally, given the small sample size and the limited ability of formal normality tests to detect departures from normality in small samples, non-parametric sensitivity analyses were performed using the Mann–Whitney U test. These analyses assessed the robustness of the results from the unadjusted parametric analyses.

Statistical analyses were performed using Jamovi software, version 2.6.45, with statistical significance set at *p* < 0.05.

## 3. Results

Regarding sample characterisation (Table 1), the study included 17 older men, divided into two groups: an aquatic physical exercise programme (AE; n = 8; 70.8 ± 4.5 years) and a multicomponent exercise programme (ME; n = 9; 75.4 ± 4.1 years). The ME group had a significantly higher mean age than the AE group (*p* = 0.040). The mean between-group difference (AE–ME) was −4.69 years (95% CI: −9.15 to −0.24), with a large effect size (*d* = −1.09; 95% CI: −2.10 to −0.05). Following the non-parametric sensitivity analysis, the between-group difference in age no longer reached statistical significance (*p* = 0.059).

For body composition parameters, the AE group showed similar mean body weight (W), slightly lower mean fat mass percentage (FM), and slightly higher mean bioelectrical impedance-derived visceral fat level (VF) and muscle mass percentage (MM) than the ME group. After checking the assumptions of normality and homogeneity of variances, an independent-samples Student’s *t*-test was applied. No statistically significant between-group differences were observed for any body composition parameter analysed: W (*p* = 0.989), VF (*p* = 0.774), FM (*p* = 0.775), and MM (*p* = 0.440).

In addition, a non-parametric sensitivity analysis was performed using the Mann–Whitney U test. The findings for the body composition parameters remained non-significant: W (U = 36.0; *p* = 1.000), VF (U = 31.5; *p* = 0.699), FM (U = 30.0; *p* = 0.606), and MM (U = 28.0; *p* = 0.481).

Regarding the mental health indicators, the AE group showed lower mean levels of self-esteem and life satisfaction compared with the ME group. Regarding depressive symptoms and perceived stress, the AE group also showed slightly lower mean values, which, on these scales, correspond to lower levels of depressive symptoms and perceived stress.

The assumption of normality was met for all variables analysed. The assumption of homogeneity of variances was met for the RSES and SWLS scales, but not for the GDS and PSS scales. Therefore, for comparisons between groups, the *p*-value from Student’s *t*-test was considered for the RSES and SWLS scales, and the *p*-value from Welch’s *t*-test was considered for the GDS and PSS scales.

In the unadjusted analysis, a statistically significant between-group difference was observed in life satisfaction, assessed using the SWLS (*p* = 0.029). This difference was associated with a large point estimate of the effect size (*d* = −1.174), although with a wide confidence interval (95% CI: −2.20 to −0.12), indicating considerable uncertainty in the estimate. The ME group showed higher mean life satisfaction than the AE group. However, given that four co-primary mental health outcomes were assessed, a multiplicity-adjusted analysis using the Bonferroni correction was performed. After this correction, the observed difference in SWLS no longer reached statistical significance (adjusted *p* = 0.116). Accordingly, this finding should be considered exploratory and interpreted with caution.

Given the statistically significant age difference between the groups, an exploratory ANCOVA was additionally performed to examine the between-group difference in life satisfaction after adjustment for age. The group effect remained statistically significant after adjustment, F(1,14) = 5.99, *p* = 0.028, *ηp^2^* = 0.30, whereas age did not show a statistically significant effect, F(1,14) = 0.59, *p* = 0.454, *ηp^2^* = 0.04. The assumptions of residual normality, homogeneity of variances, and homogeneity of regression slopes were met. Given the very small sample size, this adjusted analysis should be interpreted with caution.

No statistically significant between-group differences were observed for the remaining mental health indicators analysed: self-esteem, assessed using the RSES (*p* = 0.599; *d* = −0.261); depressive symptoms, assessed using the GDS (p = 0.890; *d* = −0.070); and perceived stress, assessed using the PSS (*p* = 0.892; *d* = −0.069). Effect sizes were considered small for the RSES and negligible for the GDS and PSS (Table 2). Because the sensitivity analysis indicated that the study could detect only very large effects (*d* ≥ 1.46), the absence of statistically significant differences in these indicators should not be interpreted as evidence of similarity or equivalence between the groups.

Given the small sample size and the limited ability of formal normality tests to detect departures from normality in small samples, a non-parametric sensitivity analysis using the Mann–Whitney U test was also performed. No statistically significant between-group differences were observed for the RSES (U = 27.5; *p* = 0.439), GDS (U = 29.0; *p* = 0.529), and PSS (U = 34.0; *p* = 0.885). For the SWLS, the parametric analysis did not reach statistical significance, and the non-parametric analysis also did not reach the threshold (U = 15.5; *p* = 0.054).

**Figure 1:**
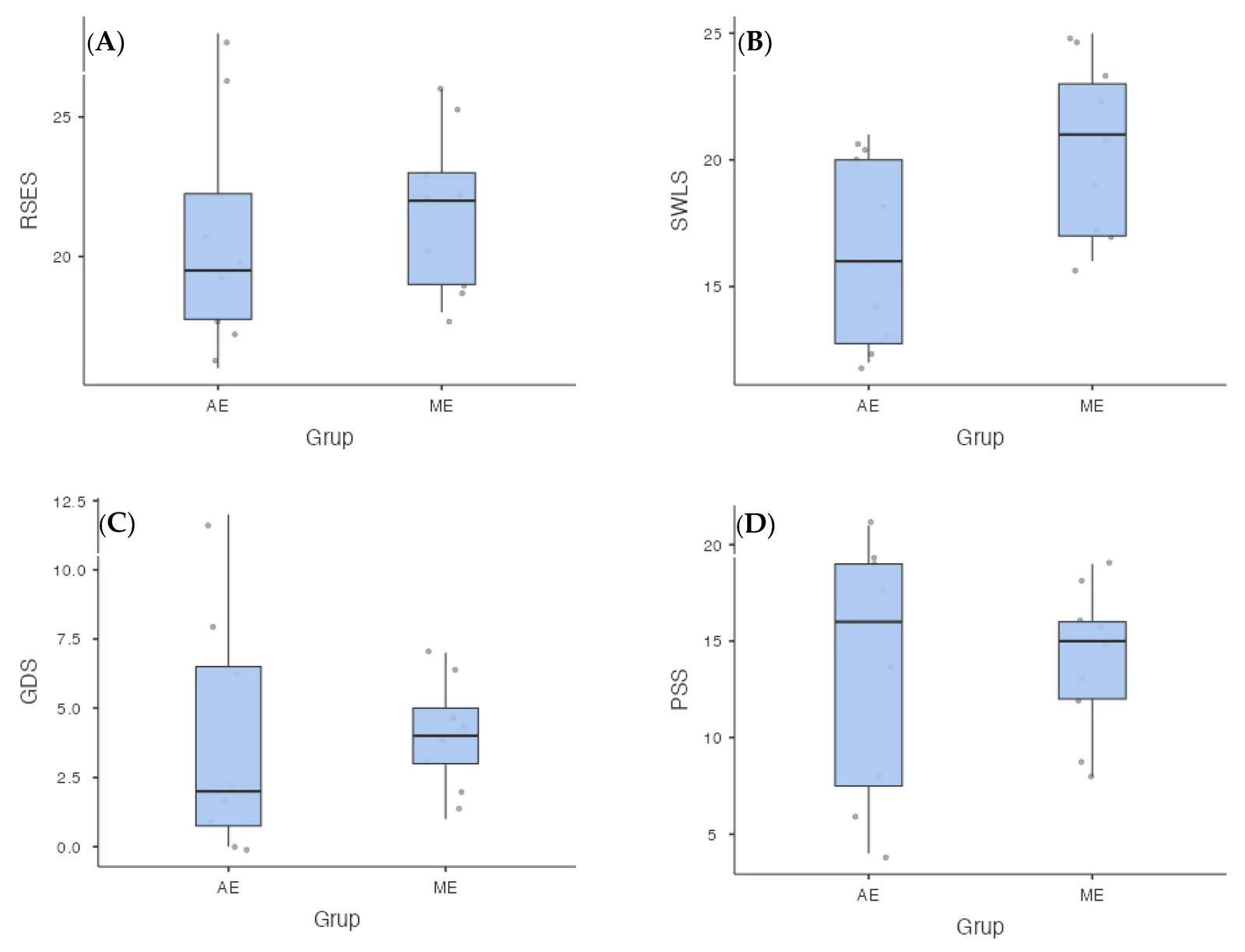
Boxplots with individual data points for the aquatic exercise (AE) and multicomponent exercise (ME) groups across the following mental health indicators: Rosenberg Self-Esteem Scale (RSES) (**A**); Satisfaction with Life Scale (SWLS) (**B**); Geriatric Depression Scale (GDS) (**C**); and Perceived Stress Scale (PSS) (**D**). The boxplots display the median and interquartile range, and the dots represent individual values.

The graphical representation of the individual data showed considerable overlap between groups across the variables analysed. However, for the SWLS, overlap was less, with values tending to be higher in the multicomponent exercise group (Figure 1).

## 4. Discussion

The present study aimed to compare mental health indicators between older men practising aquatic exercise and older men practising multicomponent exercise. The main findings showed that the multicomponent exercise group had significantly higher life satisfaction scores (unadjusted analysis), assessed using the SWLS, compared with the aquatic exercise group. This difference was associated with a large effect size estimate (*d* = −1.174); however, the corresponding 95% confidence interval was very wide (95% CI: −2.20 to −0.12), reflecting substantial uncertainty regarding the true magnitude of the effect. This uncertainty, along with limited statistical power and the loss of statistical significance in the sensitivity analyses, reinforces the need to interpret this finding as exploratory.

On the other hand, no statistically significant between-group differences were observed in self-esteem, depressive symptoms, or perceived stress. These findings should, however, be interpreted in light of the small sample size and the study’s limited statistical power. The sensitivity analysis indicated that, with the available sample, only very large effects could have been detected (*d* ≥ 1.46). Consequently, the absence of statistically significant differences in self-esteem, depressive symptoms, and perceived stress should not be interpreted as evidence of similarity or equivalence between the two exercise modalities, as smaller differences may not have been detected. In this context, the findings should be regarded as exploratory and hypothesis-generating.

In this sample, participants in the multicomponent exercise programme had higher life satisfaction scores. This finding is relevant because life satisfaction is an important dimension of quality of life and psychological well-being in older adults. In a study of men aged over 65 years, regular exercisers had higher levels of life satisfaction, self-esteem, and self-efficacy than non-exercisers [21]. The findings of the present study add preliminary evidence to the literature on the relationship between different exercise modalities and mental health indicators in older men. However, they do not establish that the observed differences resulted directly from the exercise modality practised.

The observed difference in life satisfaction may, hypothetically, be related to certain characteristics of the multicomponent exercise programme. This type of programme incorporates different components of functional fitness, such as strength, balance, coordination, flexibility, and aerobic endurance, which, according to the previous literature, may be related to perceptions of functionality, autonomy, and physical competence [9,21]. However, these mechanisms were not assessed in the present study; therefore, this interpretation should be considered only a hypothesis to be explored in future research. Previous studies indicate that multicomponent exercise may improve different domains of ageing, including physical function, cognition, frailty, and depressive symptoms; however, its effects on quality of life are not always consistent [6]. The systematic review and meta-analysis showed that structured multicomponent programmes may reduce depressive symptoms in older adults, although evidence was heterogeneous across studies and of low certainty due to factors such as risk of bias in several included studies and inconsistencies in intervention protocols [9]. Another relevant aspect in interpreting these findings is the statistically significant age difference between the groups, with the multicomponent exercise group being, on average, approximately 4.7 years older than the aquatic exercise group. This difference may have been a confounding factor that contributed to the observed findings, limiting the extent to which they can be attributed specifically to the exercise modality practised. It should also be considered that the observed difference in life satisfaction may have been influenced by other factors associated with life satisfaction that were not assessed, such as marital status, social support, clinical condition, income, educational level, and functional independence [22,23], making it impossible to isolate exercise modality as the main explanatory factor. Another consideration is that four co-primary mental health outcomes were assessed, increasing the risk of Type I error due to multiple comparisons.

Furthermore, following a multiplicity-adjusted analysis using the Bonferroni correction, the finding no longer reached the threshold for statistical significance. Accordingly, this finding should be considered exploratory and requires confirmation in studies with greater statistical power. This uncertainty is further reinforced by the non-parametric sensitivity analysis, in which the between-group difference in SWLS did not reach the conventional threshold for statistical significance (*p* = 0.054).

Regarding aquatic exercise, the literature has also reported favourable findings for older adults’ mental health, with this modality considered a viable alternative to land-based exercise. The study concluded that aquatic exercise may improve quality of life, mood, depression, anxiety, tension, and perceived efficacy regarding the risk of falling in older adults [24]. Similarly, another study found that aquatic exercise may positively affect mood and anxiety symptoms. However, the authors highlighted the need for higher-quality studies with more robust samples [4].

In this context, the present study found no statistically significant differences in self-esteem, depressive symptoms, or perceived stress. Nevertheless, given the limited statistical power, these findings do not allow the conclusion that the two exercise modalities produce similar or equivalent responses in these indicators.

Both groups comprised physically active older adults participating in regular, supervised exercise programmes. This context should be considered when interpreting the findings, as previous studies indicate that participation in regular exercise programmes is associated with better mental health indicators, lower stress, and improved quality of life in older adults [25]. However, the present study does not allow us to determine whether this characteristic shared by both groups contributed to the observed findings.

Not all studies demonstrate clear benefits across exercise modalities. A previous study found that a group-based physical activity intervention did not produce superior effects, compared with a health education intervention, on stress, fatigue, or depressive symptoms in older adults with mobility limitations [26]. Factors such as social interaction, regular participation, supervision, and the group setting may, hypothetically, influence the psychological indicators observed, as group-based exercise programmes may provide psychosocial benefits, including greater perceived social support, adherence, and improvements in some indicators of psychological well-being [27,28]. However, these dimensions were not assessed in the present study, and this possibility should be investigated in future studies.

The exclusively male sample is a relevant aspect of this study. Most studies on physical exercise and mental health in older adults include mixed-sex or predominantly female samples, which may make it difficult to interpret findings specifically in older men. This distinction is relevant, as the relationship between physical activity, physical frailty, and depressive symptoms may differ between older men and women [4,8]. Accordingly, this study provides exploratory data in an area that remains relatively understudied by directly comparing two exercise modalities frequently used in community settings in an exclusively male sample.

Future studies should include larger samples with adequate statistical power to detect smaller differences between exercise modalities, as well as a sedentary control group. It will also be important to conduct longitudinal and experimental studies to assess the effects of exercise participation over time and establish causal relationships. In addition, future research should consider further variables, such as educational level, marital status, clinical history, medication use, and sleep quality. These variables may improve understanding of the relationship between different exercise programmes and mental health indicators.

This study has several limitations. First, the small sample size substantially limits the statistical power. The sensitivity analysis indicated that, with the available sample, the study could only detect very large effects (*d* ≥ 1.46). Consequently, the absence of statistically significant differences in some indicators should not be interpreted as evidence of similarity or equivalence between the exercise modalities, as smaller differences may not have been detected. Therefore, the findings should be interpreted as exploratory and hypothesis-generating. The use of multiple comparisons across the four co-primary outcomes is also a limitation, as it may increase the risk of Type I error; in particular, the finding for life satisfaction should be interpreted with caution. Second, the cross-sectional design does not allow us to establish causal relationships between the exercise modalities practised and the mental health indicators analysed. Third, the absence of a sedentary control group prevents us from determining whether both exercise groups show better outcomes in the mental health indicators analysed compared to older men who do not engage in physical exercise. Fourth, the statistically significant age difference between the groups is a potential confounder and may have influenced the mental health indicators analysed. Fifth, the study did not collect detailed data on total participation beyond the minimum six months, physical activity outside sessions, previous exercise history, or individualised records of progression and the intensity achieved during each session. These limitations make it more difficult to characterise total exercise exposure accurately and should be considered when interpreting the findings. Sixth, although some conditions before the bioelectrical impedance assessment were standardised, the authors did not provide specific instructions on fasting or hydration status, which may have introduced variability in body composition estimates. Finally, although the comparison between exercise modalities is relevant, the groups may have differed in uncontrolled individual characteristics, such as marital status, social support, clinical condition, medication use, income, educational level, and functional independence. Furthermore, because participants were already enrolled in their respective exercise programmes before the study, self-selection could not be excluded, and it was not possible to determine whether pre-existing psychological, social, functional, or clinical differences influenced programme choice and the observed findings.

## 5. Conclusions

The findings of the present study show an exploratory difference in life satisfaction scores between the two exercise groups, with higher values in the multicomponent exercise group in the unadjusted parametric analysis. However, this finding did not remain statistically significant after correction for multiple comparisons or in the non-parametric sensitivity analysis. We observed no statistically significant between-group differences in self-esteem, depressive symptoms, or perceived stress. Given the small sample size and limited statistical power, the absence of statistically significant differences in these indicators should not be interpreted as evidence of similarity or equivalence between the exercise modalities.

Accordingly, the findings should be considered exploratory and interpreted with caution, taking into account the study’s cross-sectional design, the lack of randomisation, and potential confounding factors. Future studies with larger samples and adequate statistical power, as well as longitudinal and experimental designs, are needed to confirm these findings and to understand better the relationship between different exercise modalities and mental health indicators in older men.

## Figures and Tables

**Table 1:** Sample characteristics and body composition parameters.

	Group	Age	W (Kg)	VF	FM (%)	MM (%)
Mean	AE	70.8	80.5	15.5	24.5	33.2
	ME	75.4	80.5	14.7	25.4	32.2
Median	AE	72.0	79.2	16.0	23.1	33.9
	ME	74.0	79.6	14.0	27.2	31.5
Standard deviation	AE	4.5	10.0	4.72	4.38	2.28
	ME	4.1	9.90	6.71	7.17	2.94
Minimum	AE	65.0	69.3	10.0	19.9	28.7
	ME	68.0	65.5	4.0	11.9	28.1
Maximum	AE	76.0	97.9	23.0	32.7	35.4
	ME	82.0	95.8	28.0	34.5	37.4
25th–75th Percentiles (Q1–Q3)	AE	66.5–74.3	72.9–87.2	11.5–18.3	21.7–26.3	32.7–34.7
	ME	74.0–77.0	77.2–85.3	12.0–17.0	21.2–30.6	30.9–33.5
*p* (Shapiro–Wilk)	AE	0.187	0.479	0.441	0.291	0.119
	ME	0.718	0.780	0.857	0.635	0.682
*p* (Levene’s test)		0.548	0.698	0.671	0.198	0.480
*p* (Student’s *t*-test)		0.040	0.989	0.774	0.775	0.440
Mann–Whitney U test		16.0	36.0	31.5	30.0	28.0
*p* (Mann–Whitney U test)		0.059	1.000	0.699	0.606	0.481
Mean difference (AE-ME)		−4.69	0.07	0.83	−0.85	1.02
95% CI for Mean difference		[−9.15; −0.24]	[−10.25; 10.39]	[−5.24; 6.91]	[−7.10; 5.40]	[−1.73; 3.77]
Cohen’s *d*		−1.09	0.00	0.14	−0.14	0.39
95% CI for Cohen’s *d*		[−2.10; −0.05]	[−0.95; 0.96]	[−0.81; 1.09]	[−1.09; 0.81]	[−0.58; 1.34]

Note. AE = aquatic exercise programme (n = 8); ME = multicomponent exercise programme (n = 9); W = weight; VF = bioelectrical impedance-derived visceral fat level; FM = percentage of fat mass; MM = percentage of muscle mass.

**Table 2:** Analysis of mental health indicators.

	Group	RSES	SWLS	GDS	PSS
Mean	AE	20.6	16.3	3.88	13.6
	ME	21.6	20.6	4.11	14.0
Median	AE	19.5	16.0	2.00	16.0
	ME	22.0	21.0	4.00	15.0
Standard deviation	AE	4.3	3.9	4.4	6.7
	ME	2.8	3.5	1.9	3.8
Minimum	AE	16.0	12.0	0.00	4.00
	ME	18.0	16.0	1.00	8.00
Maximum	AE	28.0	21.0	12.0	21.0
	ME	26.0	25.0	7.00	19.0
25th–75th Percentiles (Q1–Q3)	AE	17.8–22.3	12.8–20.0	0.75–6.50	7.50–19.0
	ME	19.0–23.0	17.0–23.0	3.00–5.00	12.0–16.0
*p* (Shapiro–Wilk)	AE	0.252	0.080	0.110	0.160
	ME	0.557	0.328	0.964	0.639
*p* (Levene’s test)		0.305	0.394	0.016	0.036
*p* (Student’s *t*-test)		0.599	0.029	-	-
*p* (Welch’s *t*-test)		-	-	0.890	0.892
Bonferroni-adjusted *p*		1.000	0.116	1.000	1.000
Mann–Whitney U test		27.5	15.5	29.0	34.0
*p* (Mann–Whitney U test)		0.439	0.054	0.529	0.885
Gl		15.0	15.0	9.33	10.82
Cohen’s *d*		−0.261	−1.174	−0.070	−0.069
95% CI for Cohen’s *d*		[−1.21; 0.70]	[−2.20; −0.12]	[−1.02; 0.88]	[−1.02; 0.88]

Note. AE = aquatic exercise programme (n = 8); ME = multicomponent exercise programme (n = 9); RSES = Rosenberg Self-Esteem Scale; SWLS = Satisfaction with Life Scale; GDS = Geriatric Depression Scale; PSS = Perceived Stress Scale. Bonferroni-adjusted *p* values were calculated considering the four co-primary mental health outcomes.

## Data Availability

The data presented in this study are available on request from the corresponding author. The data are not publicly available due to the need to protect the privacy and confidentiality of the study participants.

## Abbreviations

The following abbreviations are used in this manuscript:

AE	Aquatic exercise
ME	Multicomponent exercise
RSES	Rosenberg Self-Esteem Scale
SWLS	Satisfaction With Life Scale
GDS	Geriatric Depression Scale
PSS	Perceived Stress Scale
W	Weight
VF	Bioelectrical impedance-derived visceral fat level
FM	Percentage of fat mass
MM	Percentage of muscle mass
